# Nanopore Targeted Sequencing for Rapid Gene Mutations Detection in Acute Myeloid Leukemia

**DOI:** 10.3390/genes10121026

**Published:** 2019-12-09

**Authors:** Cosimo Cumbo, Crescenzio Francesco Minervini, Paola Orsini, Luisa Anelli, Antonella Zagaria, Angela Minervini, Nicoletta Coccaro, Luciana Impera, Giuseppina Tota, Elisa Parciante, Maria Rosa Conserva, Orietta Spinelli, Alessandro Rambaldi, Giorgina Specchia, Francesco Albano

**Affiliations:** 1 Department of Emergency and Organ Transplantation (D.E.T.O.), Hematology Section, University of Bari, P.zza G. Cesare, 11 70124 Bari, Italy; cosimo.cumbo@gmail.com (C.C.); eziominervini@gmail.com (C.F.M.); paolaorsini86@gmail.com (P.O.); luisa.anelli@uniba.it (L.A.); antonellazagaria@hotmail.com (A.Z.); minervini.angela@gmail.com (A.M.); nicoletta.coccaro@uniba.it (N.C.); luciana.impera@uniba.it (L.I.); giuseppina.tota@uniba.it (G.T.); elisaparciante@libero.it (E.P.); mariarosaconserva@gmail.com (M.R.C.); specchiagiorgina@gmail.com (G.S.); 2 Hematology and Bone Marrow Transplant Unit, University of Milan, 24127 Bergamo, Italy; ospinelli@asst-pg23.it (O.S.); alessandro.rambaldi@unimi.it (A.R.)

**Keywords:** nanopore targeted sequencing, acute myeloid leukemia, mutational analysis, *FLT3* internal tandem duplications, biallelic *CEBPA* mutations

## Abstract

Acute myeloid leukemia (AML) clinical settings cannot do without molecular testing to confirm or rule out predictive biomarkers for prognostic stratification, in order to initiate or withhold targeted therapy. Next generation sequencing offers the advantage of the simultaneous investigation of numerous genes, but these methods remain expensive and time consuming. In this context, we present a nanopore-based assay for rapid (24 h) sequencing of six genes (*NPM1*, *FLT3*, *CEBPA*, *TP53*, *IDH1* and *IDH2*) that are recurrently mutated in AML. The study included 22 AML patients at diagnosis; all data were compared with the results of S5 sequencing, and discordant variants were validated by Sanger sequencing. Nanopore approach showed substantial advantages in terms of speed and low cost. Furthermore, the ability to generate long reads allows a more accurate detection of longer *FLT3* internal tandem duplications and phasing double *CEBPA* mutations. In conclusion, we propose a cheap, rapid workflow that can potentially enable all basic molecular biology laboratories to perform detailed targeted gene sequencing analysis in AML patients, in order to define their prognosis and the appropriate treatment.

## 1. Introduction

Acute myeloid leukemia (AML) is a molecularly heterogeneous hematological malignancy with a variable prognosis and response to treatment [1,2]. Recurring molecular lesions identify AML patient subgroups with different survival probabilities; in fact, results from *nucleophosmin 1* (*NPM1*), *Fms-like tyrosine kinase 3* (*FLT3*), *CCAAT/enhancer binding protein α* (*CEBPA*) and *tumor protein p53* (*TP53*) mutational screening genes have led to recommendations from the European LeukemiaNet Group that these be tested in routine practice [3]. Moreover, since the United States Food and Drug Administration (FDA) approval of targeted inhibitors for *FLT3*, *isocitrate dehydrogenase 1* (*IDH1*) and *2* (*IDH2*) gene mutations, predictive biomarkers are also now needed to select AML patients for targeted therapy [4,5,6]. 

Therefore, in AML clinical settings, molecular testing is a requisite in laboratories to confirm or rule out predictive biomarkers, in order to initiate or withhold targeted therapy, or to enroll patients in specific clinical trials once their prognostic risk has been defined. In this context, assays performed by massive parallel next-generation sequencing (NGS) offer substantial advantages in the form of a simultaneous investigation of numerous genes. However, the cost of NGS-based methods is still high, in terms of both capital expenses and expertise. Moreover, NGS turnaround time is also quite long, reaching up to 7 days. In fact, depending on the type of test and the instrument used, the assay time is contingent on library preparation, sequencing and downstream analysis, but it may be further prolonged by the need to batch samples so as to reduce costs and workload. New sequencing platforms, such as the Oxford Nanopore MinION system, offer the possibility of rapid sequencing and immediate availability of data for analyses. Nanopore sequencing is characterized by long reads and real-time data generation, features that make it an ideal tool for targeted genes sequencing [7]. MinION has already been successfully used by our group to detect mutations of the *TP53* and *ABL1* genes in chronic lymphocytic leukemia (CLL) and in chronic myeloid leukemia (CML) patients, respectively [8,9,10]. Moreover, we recently described the development of a customized, MinION-based gene panel for the targeted sequencing of genes recurrently mutated in CLL, demonstrating a satisfactory performance [11]. 

Herein, we describe a nanopore-based assay for the rapid sequencing of six genes (*NPM1*, *FLT3*, *CEBPA*, *TP53*, *IDH1* and *IDH2*) that are recurrently mutated in AML. NGS blinded gene analysis was performed on the specimens collected.

## 2. Materials and Methods

### 2.1. Patients

Twenty-two acute myeloid leukemia (AML) patients were included in this study (Table S1). Molecular evaluation [i.e., *NPM1* (A or B mutations) and *FLT3* internal tandem duplications (ITD) or tyrosine kinase domain (TKD) mutations] was performed at diagnosis in all cases. Among them, we selected five patients (AML#18-AML#22) harboring a complex karyotype, in an effort to increase the chances of finding cases with the *TP53* gene mutations, in accordance with what has already been reported [12]. No data were available in our series for the *CEBPA*, *IDH1* and *IDH2* gene mutational status. In all the AML cases included in this work, recurrent chromosomal rearrangement was excluded by molecular and fluorescence in situ hybridization experiments with specific bacterial artificial clones, as previously described [13,14,15,16]. The AML cases were subdivided into two groups, and for the purposes of the barcoding process, each group had to include no more than 12 samples. Therefore, each group consisted of 11 cases (AML#1–AML#11 in the first and AML#12–AML#22 in the second) and one negative healthy control sample (NC) (Human CEPH Genomic DNA Control by Thermo Fisher Scientific Waltham, MA, USA). Genomic DNA (gDNA) was extracted from bone marrow mononuclear cells, isolated by Ficoll density centrifugation, using the QIAamp DNA Blood Mini Kit (Qiagen, Hilden, Germany) and quantified with the Qubit 2.0 Fluorometer (Life Technologies Carlsbad, CA, USA). The study was approved by the local ethics committee “Azienda Ospedaliero Universitaria Policlinico di Bari” No. 624 from 21 May 2010. Written, informed consent was obtained from all patients before enrolment in accordance with the Declaration of Helsinki. AML patient records/information were anonymized and deidentified prior to analysis. 

### 2.2. Acute Myeloid Leukemia Panel Design and Testing

Our customized AML gene panel for MinION included the known mutation hotspots of *NPM1* (*exon 11*), *FLT3* (*exons 14,15,20*), *IDH1* (*exon 4*) and *IDH2* (*exon 4*); *CEBPA* (*full gene*) and *TP53* (*exons 2–11*). To enrich these genomic regions, we adopted a polymerase chain reaction (PCR)-based strategy using a combination of primers selected from a larger customized AML panel, designed with the Ion AmpliSeq Designer tool (Thermo Fisher Scientific), used for data validation. 

Overall, 10 pairs of primers were selected for the target genes and analyzed with the Multiplex 2.1 tool (http://bioinfo.ebc.ee/multiplx) to evaluate primers’ compatibility and find the best primers pooling solution. In detail, we chose to consider any primer–primer interactions and to pull in the same group amplicons with a maximum length difference of 400 bps. Three primers pools were thus identified: pool 1 included primers for *CEBPA exon 1*, *TP53 exons 7–9* and *TP53 exons 10–11*. Pool 2 was assembled with primers for *NPM1 exon 11*, *FLT3 exons 14–15*, *FLT3 exon 20* and *IDH2 exon 4*. Finally, pool 3 included primers for *TP53 exons 2–4*, *TP53 exons 5–6* and *IDH1 exon 4*. The total panel size was about 7 kb. Table 1 shows the composition of the three primer pools, the respective amplicon sizes and the primers sequences.

### 2.3. Multiplex Long-Polymerase Chain Reaction

For each sample, three multiplex long-PCRs were performed using the PrimeSTAR GXL DNA Polymerase (Takara Bio Inc., Shiga, Japan), 100 ng of gDNA, in a final volume of 50 μL. Thermal-cycling conditions were 98 °C for 10 s, 55 °C for 15 s, 68 °C for 90 s (30 cycles) and 4 °C hold for pool 1; 98 °C for 10 s, 60 °C for 15 s, 68 °C for 60 s (30 cycles) and 4 °C hold for pool 2 and pool 3. The PCR products were visualized by SYBR Safe on an agarose-gel. Since two amplicons in pool 2 had a very similar size of about 220 bps and were not easily distinguishable by 2.2% agarose gel electrophoresis, a restriction enzyme digestion of these critical amplicons was made, and the EcoRV-HF restriction enzyme (20,000 units/Ml; New England BioLabs Inc., Ipswich, MA, USA) was finally selected to verify their successful amplification and to discriminate them. Twenty uL of the primer pool 2 PCR products were incubated with 20 units of EcoRV-HF in CutSmart Buffer for 1 h at 37 °C. Digestion products were loaded on a 2.2% agarose gel. To equalize the amount of amplicons contained in each pool, we evaluated the intensity of bands visualized by SYBR Safe on agarose gel. In detail, we increased by four-fold the concentration of the primers for *NPM1 exon 11* and *FLT3 exon 20* in pool 2 and halved the concentration of the primers for *TP53 exon 2–4* in pool 3. The PCR products were purified using the QIAquick PCR Purification Kit (Qiagen). Before starting library preparation, we quantified and estimated the purity of samples (Nanodrop, Thermo Fisher Scientific). Equimolar amounts of the three PCR products (calculated on the average length of the amplicons for each pool) were mixed.

### 2.4. MinION Sequencing (MS)

According to the 2D Native barcoding genomic DNA (SQK-LSK 208) protocol, a total volume of 25 uL, containing 500 ng of the amplicons, was end-prepared using the NEBNext Ultra II End Repair/dA-Tailing Module (New England Biolabs Inc.) and barcoded with the ligation of nanopore-specific Native Barcodes (NB01-NB12) using Blunt/TA Ligase Master Mix (New England Biolabs Inc.). Equimolar amounts of each barcoded amplicon were then pulled. According to the Ligation Sequencing Kit 1D (SQK-LSK108) protocol, a total volume of 50 uL containing 700 ng of the barcoded amplicons was prepared for sequencing. After the Platform QC run and the priming of the flowcell, the sequencing mix was loaded and the NC_48Hr_sequencing_FLO-MIN107_SQK-LSK108 protocol was started (MinIONflowcell: FLO-MIN107).

### 2.5. MinION Sequencing Data Analysis

The fast5 files resulting from the sequencing were uploaded in Albacore (v.2.3.3) for base-calling and demultiplexing. The NanoOK tool (stable version) was employed for coverage and error assessment, using the FASTA sequences of the target amplicons as reference. Reads were aligned on the GRCh37 human reference genome with the BWA–MEM method [17] using specific Nanopore platform parameters and visualized with the Integrative Genomics Viewer (IGV) browser [18]. For each patient, variant calling was performed with the Somatic Mutation Calling tool, Varscan 2.4.3 (https://github.com/dkoboldt/varscan/). The ‘SNV’ (single-nucleotide variant) and ‘INDEL’ (insertion-deletion) files were filtered as reported below. Data were then annotated for refGene, exac03, avsnp150 and cosmic81 databases using Annovar software tool (http://annovar.openbioinformatics.org/en/latest/) and filtered for exonic/intronic position and mutation effect. All results from MinION Sequencing (MS) were finally compared with the results from S5 sequencing, (S5S) and discordant variants were validated by Sanger Sequencing (SS). 

To detect samples with *FLT3* ITD from MS we used the specific tool, Sniffles [19]. The samples that were positive for the ITD were further analyzed to extract the consensus sequences of the tandem repeat. The FASTA sequences of the reads mapping on the *FLT3* ITD region were extracted from the bam files and were used to assemble the ITD sequence using the CAP3 [20] algorithm, that produced some consensus sequences. All consensus sequences were then multialigned together with the reference sequence. At the end, the inserted repeats were easily detected from the multialignment file. The complete pipeline with command lines and parameters is reported in File S1. 

For the AML cases harboring two *CEBPA* mutations each, the reads covering the whole genomic interval between the *CEBPA* variants identified and without any mismatches or deletions in the genomic sequences flanking these variants, were filtered with “samtools view” command and samjs tool (Jvarkit) and used for phasing. From these filtered BAM files, the reads with the SNV/small INDELs called were selected with the “samtools view” command, whereas the VariantBam tool was applied to operate a less stringent selection of the reads harboring the larger *CEBPA* INDEL identified. The resulting filtered bam files were visualized in the IGV software. From the corresponding SAM files, the identifiers of the reads supporting each *CEBPA* mutation were retrieved, and the two lists of reads were compared in a Venn diagram, in order to establish the relationship between the two *CEBPA* mutations.

MinION Sequencing data analysis was conducted on Intel^®^ Core^TM^ i7-7700K CPU @ 420GHz RAM16GB IO Unity SSD 1TB (ASUS, Beitou District, Taipei, Taiwan).

### 2.6. S5 Sequencing (S5S)

Ten ng of the same samples used for MS were analyzed by S5S. A customized panel, encompassing the full coding regions or specific exons of 26 target genes involved in the pathogenesis of myeloid malignancies pathogenesis, was used; library preparation and data analysis were performed as previously reported [21]. The data analysis was focused only on the genomic regions included in our customized panel used for MS. The choice of the S5S strategy derives from the need to compare MS results with a conventional NGS approach. However, it must be specified that the two platforms differ, not only for the sequencing technology (nanopore-based for MinION and semiconductor-based for S5), but also for the library preparation chemistry: direct amplicon sequencing vs. clonal amplification by emulsion-PCR and direct DNA strands reading vs. sequencing by synthesis, respectively.

### 2.7. Sanger Sequencing (SS)

For SS, we performed a PCR target enrichment of the genomic regions for which MS and S5S produced discordant results. For *FLT3* ITD gene analysis, we used Platinum Taq DNA Polymerase (Invitrogen, Carlsbad, CA, USA), with 200 ng of gDNA, two primers as previously described [22], in a final volume of 50 uL. Thermal cycling conditions were: an initial denaturation of 95 °C for 3 min, 95 °C for 30 s, 62 °C for 30 s, 72 °C for 30 s (35 cycles), 72 °C for 5 min and 4 °C hold. For *TP53* we used the International Agency for Research on Cancer (IARC) protocol (http://p53.iarc.fr/). For *CEBPA* we used PrimeSTAR GXL DNA Polymerase (Takara Bio Inc.), 100 ng of gDNA, two primers (*CEBPA*_179F:CGTCCATCGACATCAGCGCCTA,*CEBPA*_521R: GCCAGCTGCTTGGCTTCATCCT ) in a final volume of 50 μL. Thermal-cycling conditions were 98 °C for 10 s, 55 °C for 15 s, 68 °C for 1 min (30 cycles) and 4 °C hold. The PCR products were visualized on a 2% agarose gel, the bands were sliced, purified using the QIAquick PCR Purification Kit (Qiagen), quantified with a Qubit 2.0 fluorometer (Life Technologies) and prepared for SS. Electropherograms were then analyzed by visual inspection with the FinchTV software (v.1.4.0; Informer Technologies, Inc.). For the *FLT3* ITD analysis, we used the tool Indigo.

### 2.8. Data Availability

The sequence data from this study have been submitted to the National Center for Biotechnology Information (NCBI) Short Read Archive (https://www.ncbi.nlm.nih.gov/sra/) under accession Number PRJNA527949.

## 3. Results

### 3.1. MinION Sequencing Performance Evaluation 

**Figure 1:**
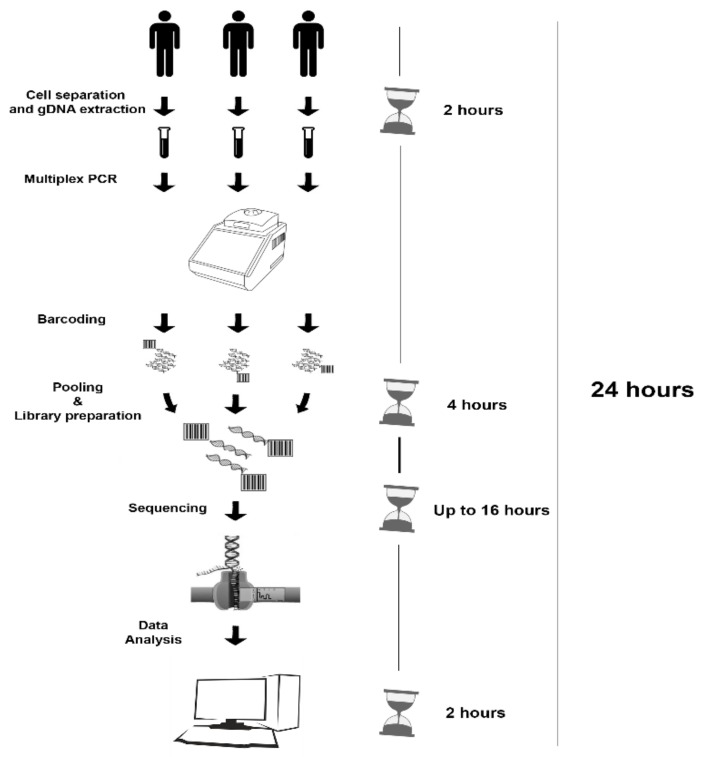
Schematic workflow implemented for MinION sequencing approach.

In 24 h from sample collection (cell separation and gDNA extraction: 2 h, multiplex long-PCR and library preparation: 4 h, sequencing run: up to 16 h or overnight, data analysis: 2 h) the results from MS were obtained (Figure 1). 

Two MS runs were performed, employing two libraries of 11 patients plus an NC for each and two different flowcells, with 1085 and 1006 active pores, respectively. A total of 4,556,387 fast5 files containing raw electric signals was produced. The fast5 files were uploaded in Albacore for base-calling and demultiplexing, producing about 1.2 Gb. Finally, of the total reads produced, 1,280,642 had a recognizable barcode. The mean reads length was 950 bp, corresponding to the expected amplicons size. NanoOK analysis was performed to calculate the error rate for each amplicon. Table 1 shows the mean values of identity per 100 aligned bases and the mean error rate for insertions, deletions and substitutions for each amplicon. As observed, separate error rate analysis for insertions, deletions and substitutions revealed higher error values for deletions, in line with historically known nanopore error rate data [23,24,25]. Coverage analysis showed that all genomic regions included in our gene panel were completely covered in each sample. As reported in Table S2, for each amplicon the minimum sequencing depth value was never below 50×, except for *NPM1* in cases AML#4, AML#7, AML#14 and AML#18.

### 3.2. Variant Calling, Filtering and Annotation

The main drawback of using MinION in variant analyses is its error rate. In our experience, data from MS is affected by two kinds of errors, sporadic and recurrent, which require ad hoc filtering strategies. Generally, sporadic errors are easily overcome by increasing the depth of coverage, but reducing the sensitivity in reporting variants is required. For this reason, we set the minimum variant allele frequency (VAF) to 10% for SNV and to 15% for INDELs in variant filtering [8,11]. On the other hand, recurrent errors are easily detectable in more than one sample, but are characterized by a homopolymeric genomic context and a high VAF value, which does not allow a reasonable threshold to discriminate real variant from the sequencing error to be set. 

Our past strategy to manage recurrent errors was to exclude affected positions from the analysis, thereby reducing the breadth of coverage of the panel [11]. In this study, we developed a pipeline to better manage the error effect in the final result, using the “Somatic Mutation Calling” of Varscan (version 2.4.3) in order to compare the variants called in two NCs to each case. In our pipeline, when the same variant is called both in the tumor and the NC, the significance of allele frequency difference is calculated by Fisher’s Exact Test. SNV and INDEL files obtained from the previous variant calling step were further filtered by VAF difference in NC and tumor (>10%), considering only variants featuring a VAF > 7% in the NC (this cut-off value corresponded to the maximum mean sequencing error rate calculated for the amplicons of our gene panel, see Table 1). A second filter was applied on *p*-values, filtering off variants with a *p*-value > 0.01 (Filtering R script has been supplied in the File S2). Variant calling, filtering and annotation produced a total of 34 variants, excluding *FLT3* ITD. Among them, ten were discordant and needed validation by SS. Overall, MS identified a false positive variant: an insertion in *TP53* gene at the end of a long homopolymer sequence (locus: *chr17:7579470*) (this type of error has already been described [11]). The only false negative result from MS was a *NPM1* mutation (locus: *chr5:170837543*) due to a very low depth of coverage. On the other hand, S5S failed to detect two small INDELs on the *CEBPA* gene (loci: *chr19:33793152* and *chr19:33793082*). As reported in Table 2, the VAFs of discordant variants detected on the *CEBPA* gene in loci *chr19:33792277*, *chr19:33792729* (recurring 3 times) and *chr19:33792731* (recurring twice) are below the SS sensitivity. Anyway, even if we cannot certainly clarify this discrepancy, considering the good quality of the electropherograms obtained, the recurrence of the variants and the known problematic nature of *CEBPA* sequencing, these discordant variants could be reasonably considered as false positive. The specific error rate associated to all MS variants identified with the pipeline developed was also determined and verified by visual inspection of BAM files in both of the NCs sequenced. Overall, as reported in Table S3, the mean error rate associated with these specific variants was significantly below the VAF cut-off of 7% except for two INDELs and one SNV, harboring a higher error rate. A specific analysis of the variants detected in the two NCs was also performed on *CEBPA* and *TP53*, the two genes entirely covered in our assay, in order to identify SNVs and INDELs called with a VAF above 10% and 15%, respectively (Table S4). The two NCs were analyzed in an independent manner, identifying a total of 12 SNVs and 41 INDELs; the variants called only in a single NC had a VAF value close to the cut-off used for variant filtering (10% and 15% for SNVs and INDELs, respectively). For these reasons, as currently these genomic positions cannot be analysed in a robust manner, the final breadth of coverage of *TP53* was 99.3%, as compared to the previous MS performances [11], and for *CEBPA* it was 98.3%. These preliminary results are intended to improve with the concomitant refinement of nanopore sequencing and the dedicated analysis tools.

### 3.3. Identification of the Acute Myeloid Leukemia Hotspot Mutations

**Figure 2:**
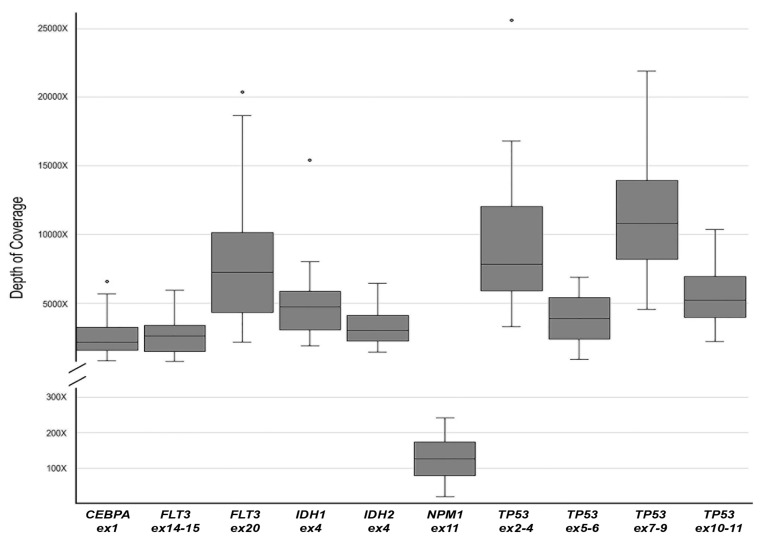
Boxplot of MinION sequencing depth of coverage, calculated for each amplicon.

We focused on the following AML hotspot mutations: *NPM1* p.W288fs, *FLT3* p.D835, *IDH1* p.R132, *IDH2* p.R140 and p.R172. For all these variants there was an almost complete concordance between data obtained from MS and S5S (Table 2). In our cohort of AML cases, we detected a total of 14 hotspot mutations: seven carrying the *NPM1* p.W288fs variant, one case with the *FLT3* p.D835 variant, two cases showing the *IDH1* p.R132 variant and four cases carrying the *IDH2* p.R140. The sole difference between MinION and S5 results was found in case AML#7 for *NPM1*. As reported in the coverage analysis (Figure 2) of the MinION data, almost all targets were well covered (>500×), excluding *NPM1* that showed a general lower coverage. In particular, case AML#7 showed a depth of coverage on *NPM1* below 50×, that caused the failure to detect the mutation. However, despite the lowest coverage observed in the MS analysis of the *NPM1* gene, in all other AML cases the comparison between MS and S5 not generated false positive or negative results related to *NPM1* gene status, and MS results were confirmed by S5S. We have not identified the cause of this state that does not appear to be related to the amplicon size nor to the nucleotide sequence of the genomic region, but that could link to the nature of multiple gene analysis. Further improvements are needed to overcome this issue by redesigning and testing new primer sets or adopting alternative enrichment strategies (i.e., hybridization [26] or Cas9 [27] methods).

### 3.4. FLT3 Internal Tandem Duplications (ITD) Data Analysis

Using the Sniffles tool, since Varscan was not able to detect large INDELs, in total, nine patients were identified as ITD positive (Figure S1). All ITDs (median length 48 bp min.–max 30–165 bps) were detected from MS and were confirmed by a specific PCR assay [22]. Results from S5S, instead, returned AML#4 as false negative (ITD was length 165 bps). In particular, the false negative resulting from the S5 analysis could be due to the length of the insertion/duplication and then to the limit of the read length of the NGS. Furthermore, in AML the size of *FLT3* ITD is very variable. 

It is now known that longer *FLT3* ITDs are associated with a higher *FLT3* kinase activity and worse outcome [28,29,30]; however, the detection of long ITD and the detection of ITDs in combination with deletions remain challenging when performed by short-reads-based technologies [31]. MinION data do not have this limit, and for this reason resulted more suitable for this purpose.

### 3.5. CEBPA Data Analysis

**Figure 3:**
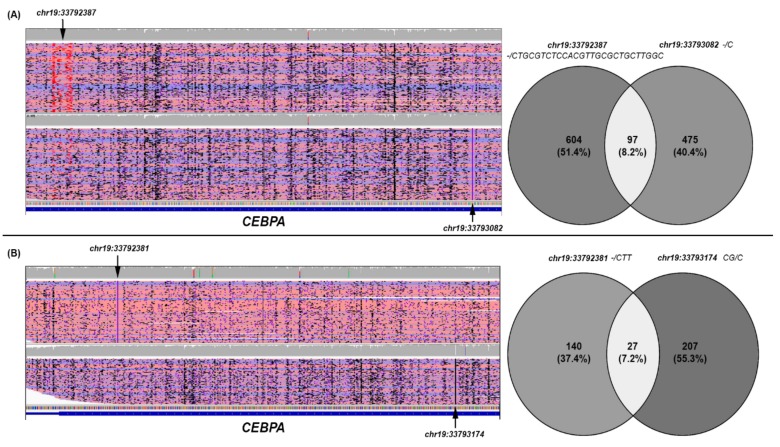
Data analysis of *CEBPA* MinION Sequencing in AML#13 (**A**) and AML#14 (**B**). For both cases a snapshot is shown of the filtered alignments, visualized with the IGV tool and supporting each *CEBPA* variant detected (left) and the Venn diagram of the reads identifiers supporting them (right). The black arrows indicate the specific variant supported in each filtered alignment; according to IGV display options, insertions larger than 20 nucleotides are flagged in red (**A**), whereas smaller insertions are indicated with a purple flag (**A**,**B**). As regards the Venn diagrams, for both cases, the identifiers of the reads supporting each *CEBPA* mutation were retrieved from the corresponding filtered SAM files, and visually compared to establish the number of reads supporting the variants detected and phase the two *CEBPA* mutations.

For AML#13 and AML#14 cases bearing two *CEBPA* mutations, each detected by MS, the reads containing the two variants were extracted from the original bam files. Overall, comparing the reads containing one or the other of the two *CEBPA*-detected variants, in both AML cases, most reads had one of the two identified mutations (91.8% and 92.7%, respectively), whereas only a small fraction of reads contained both of them (8.2% and 7.2%, respectively) (Figure 3). 

This observation indicated that in these AML patients the two *CEBPA* mutations detected in each case were predominantly located on different alleles; however, this cannot exclude the possibility that they occurred in different cells. As regards the co-occurrence of both *CEBPA* mutations in a small subset of reads, this circumstance must presumably be explained by MinION context-specific error, frequently associated with homopolymer sequences, or in the case of larger insertions/duplications, the detection of similar events in the flanking genomic regions, rather than by the GC content characteristic of *CEBPA* [32]. Mutations in the *CEBPA* gene occur in 7–15% of all AML cases; the subgroup of biallelic *CEBPA* mutations in AML patients has now been acknowledged as a definite entity in the recent World Health Organization (WHO) classification, given its distinct biological and clinical features, as well as its prognostic significance [1]. To date, conventional capillary sequencing has been the gold-standard DNA sequencing technique [33]. 

Because of the lack of appropriate coverage across the entire gene and of the high GC content leading to suboptimal amplification efficiency, a poor coverage and read depth in this gene using NGS has been reported [34,35]. Our analysis revealed that *CEBPA* gene sequencing by S5S generated false positives whereas MS is more precise and also more concordant with SS results. Moreover, the ability to generate long reads spanning the entire gene allows AML cases with biallelic *CEBPA* mutations (Figure 3) to be easily highlighted and phased, as described in previous studies concerning other genes or genomic rearrangements [36,37] without completely excluding the possibility that they may occur in different leukemic cells, our results are also consistent with a milestone study focused on *CEBPA* double mutations studied by cloning [33]. On the other hand, our analysis showed that *CEBPA* gene sequencing performed by MS is associated with nanopore context-specific error, frequently associated with homopolymeric sequences; this circumstance does not affect the integrity of the previously described result.

## 4. Discussion

The identification of gene mutations in AML patients has become routine in molecular diagnostic laboratories via a variety of techniques. Many gene panels with varying sizes were developed in the last years with the aim of providing essential information for the prognostic definition and for the therapeutic management of AML patients, and recently it was validated a 19-gene AML-targeted NGS panel that could be a valid approach to obtain clinically relevant information [38]. All conventional NGS approaches are affected by two main limits: a quite long turnaround time (TAT) reaching up to seven days, and the read length. Our work shows that the long-reads nanopore sequencing approach for gene mutation analysis in AML is feasible and potentially able to satisfy the need of reducing the TAT for NGS analysis. Nowadays, it is known that the complete validation of NGS tests includes several topics, such as the use of reference cell lines or materials for the evaluation of assay performance, the assessment of sequencing metrics and the optimum number of samples for test validation. According to the regulatory requirements of Clinical Laboratory Improvement Amendments for laboratory-developed tests, the evaluation of accuracy, precision, analytical sensitivity/limits of detection, analytical specificity and the use of sensitivity controls for the detection of targeted mutations at the lower limit of detection (LLOD) need to be defined [39]. Recommendations for an analytical validation of NGS bioinformatics pipelines to be used for clinical detection of gene variants are also available [40]. In our work we started with a relatively small set of samples to evaluate the potential of MS in the context of AML molecular analysis, especially in relation to specific genomic issues which are difficult to solve with conventional NGS strategies. However, given the satisfactory results obtained, when the technology will be ready for its use in the clinical context, a validation set will need to corroborate MinION performances. In the last decade, Illumina and Ion Torrent sequencers have been extensively evaluated and validated in the clinical setting, whereas MinION sequencer was launched in pre-release form in 2014 and today is an appealing, new sequencing paradigm continuously improving in terms of analytical performance [23,41]. Nowadays, Nanopore sequencing is still in its testing phase, even if an increasing improvement in performance compared to the beginning has been widely documented. On the other hand, conventional NGS technologies are extremely powerful, but they also have some drawbacks. One major limitation is the generation of short reads. The sequencing of the long-repeated sequences of the human genome may lead to misassemblies and gaps. In addition, large structural variations are more challenging to detect and characterize, using short reads. Moreover, NGS methods based on PCR enrichment show difficulties for GC-rich regions. The nanopore sequencing approach overcomes these difficulties; this is a very important aspect especially in the context of AML, where *FLT3* ITD and *CEBPA* mutations detection by NGS is complicated. Another main advantage highlighted in our work is that reliable and absolutely reproducible results regarding the mutational status of the *FLT3*, *IDH1* and *IDH2* genes, where mutations have an established therapeutic indication (midostaurin, enasidenib and ivosidenib, respectively), can be obtained in a very short time, about 24 h from sample collection.

## 5. Conclusions

Reducing the TAT is crucial in AML management, as recently described in the Patel et al. study, in which a custom platform designated as Ultra-rapid Reporting of GENomic Targets (URGENTseq) is reported [42]. Through the NGS workflow optimization and innovative custom bioinformatics pipeline, the platform allows the analysis of selected genes useful for diagnosis and treatment decisions in hematologic malignancies within 48 h of specimen collection [42]. We propose a workflow that can potentially enable laboratories equipped with only basic molecular biology techniques to perform detailed targeted gene sequencing analysis in AML patients, shortening TAT and reducing costs (minimal IT infrastructure for sequencing and data analysis: Windows/OSX/Linux operative systems, 16 GB RAM, i7 or Xenon with 4+ cores CPUs, 1 TB internal SSD storage unity, USB3 ports). In fact, processing the sample in the immediate onset of the disease, the cost of the analysis is around USD 200. Moreover, the MinION cost (USD 1000) could enable all basic molecular biology laboratories to perform detailed targeted gene sequencing analysis in AML patients without renouncing the quality of the results that, in the context of AML onset, is crucial to define the prognosis and treatment. Furthermore, scalability is one of the emerging strengths of nanopore technology; in fact the platform can be adapted for smaller, frequent and rapid sequencing runs using a single-use flowcell, delivering up to 1.8 Gb of data (https://nanoporetech.com/products/flongle). The preliminary results about sequencing performances of our assay, together with the constant improvement of nanopore sequencing technology, pave the way for its future application in AML diagnostics.

## Figures and Tables

**Table 1:** Composition of the three pools of the customized acute myeloid leukemia (AML) gene panel, with the genomic region covered, the primer sequences and the size of the corresponding amplicons. For each amplicon, the error rate analysis of MinION sequencing data is shown.

Pool	Target	Genomic Region	Length (bp)	Primers	Identical Bases Per 100 Aligned Bases	Inserted Bases Per 100 Aligned Bases	Deleted Bases Per 100 Aligned Bases	Substitutions Per 100 Aligned Bases
1	*CEBPA exon 1*	*chr19:33792118-33793481*	1363	5′-GGAGGCACCGGAATCTCCTA-3′ 5′-GGCCTGCCGGGTATAAAAGCTG-3′	86%	2.9%	5.6%	5.4%
	*TP53 exons 7–9*	*chr17:7576463-7577667*	1204	5′-CAGGCTAGGCTAAGCTATGATGTTCCTTAGA-3′ 5′-CTTGCCACAGGTCTCCCCAAGG-3′	87.8%	2.5%	5.6%	4%
	*TP53* *exons 10–11*	*chr17:7572810-7574095*	1285	5′-GTGCTTCTGACGCACACCTATTG-3′ 5′-AACCATCTTTTAACTCAGGTACTGTGT-3′	87.5%	2.5%	5.9%	4.1%
2	*NPM1* *exon 11*	*chr5:170837410-170837635*	225	5′-GTTAACTCTCTGGTGGTAGAATGAAAAATAGA-3′ 5′-GATATCAACTGTTACAGAAATGAAATAAGACG-3′	89.3%	2.4%	5.4%	2.9%
	*FLT3* *exons 14–15*	*chr13:28607916-28608407*	491	5′-GGCAAACAGTAACCATTAAAAGGATGG-3′ 5′-TTCCTCTATCTGCAGAACTGCCTA-3′	88.5%	3.3%	4.6%	3.6%
	*FLT3* *exon 20*	*chr13:28592521-28592740*	219	5′-CACAGTGAGTGCAGTTGTTTACCA-3′ 5′-GTCATTCTTGACAGTGTGTTCACAG-3′	90.2%	2.7%	3.4%	3.7%
	*IDH2* *exon 4*	*chr15:90631713-90632029*	316	5′-CACAAAGTCTGTGGCCTTGTACT-3′ 5′-GTTGAAAGATGGCGGCTGCA-3′	88.8%	2.3%	5.4%	3.6%
3	*TP53* *exons 2–4*	*chr17:7579204-7579986*	782	5′-GAAGCCAAAGGGTGAAGAGGAATCCC-3′ 5′-AGGGTTGGAAGTGTCTCATGCTGGA-3′	86.4%	2.2%	7.3%	4%
	*TP53* *exons 5–6*	*chr17:7578016-7578702*	686	5′-TTCAACTGTGCAATAGTTAAACCCAT-3′ 5′-CTGAGGTGTAGACGCCAACTCTC-3′	87.9%	2.6%	5.2%	4.2%
	*IDH1* *exon 4*	*chr2:209113021-209113452*	431	5′-ATACAAGTTGGAAATTTCTGGGCCAT-3′ 5′-CACTGCAGTTGTAGGTTATAACTATCCA-3′	88.9%	2.3%	4.8%	3.7%
**Mean**					88.1%	2.6%	5.3%	3.9%

**Table 2:** Description of variants identified by MinION sequencing and S5 sequencing. Only discordant variants were validated by Sanger sequencing.

Case	Gene	Locus	Genotype	Coding Change	Amino Acid Change	Type	Function	Length	Exon	MinION (VAF %)	S5 (VAF %)	Sanger
**AML#1**	*IDH1*	*chr2:209113113*	*G/A*	c.394C	p.R132	SNV	missense	1	4	YES (48.9)	YES (47.1)	N/E
**AML#2**	*FLT3*	*chr13:28608329*	*A/C*	c.1727T > G	p.L576R	SNV	missense	1	14	YES (14.4)	YES (8.3)	N/E
**AML#3**	*NPM1*	*chr5:170837547*	*-/TCTG*	c.859_860insTCTG	p.W288fs*12	INDEL	Frameshift Insertion	4	11	YES (35.3)	YES (54.5)	N/E
**AML#4**	*IDH1*	*chr2:209113113*	*G/A*	c.394C	p.R132	SNV	missense	1	4	YES (16.9)	YES (14.4)	N/E
**AML#5**	*NPM1*	*chr5:170837547*	*-/TCTG*	c.859_860insTCTG	p.W288fs*12	INDEL	Frameshift Insertion	4	11	YES (56.5)	YES (45.8)	N/E
	*IDH2*	*chr15:90631934*	*C/T*	c.419G	p.R140	SNV	missense	1	4	YES (32.4)	YES (51.3)	N/E
**AML#7**	*NPM1*	*chr5:170837547*	*-/TCTG*	c.859_860insTCTG	p.W288fs*12	INDEL	Frameshift Insertion	4	11	NO	YES (58.5)	YES
	*IDH2*	*chr15:90631934*	*C/A*	c.419G	p.R140	SNV	missense	1	4	YES (38.6)	YES (53.5)	N/E
	*CEBPA*	*chr19:33792277*	*G/CT*	c.1044delinsAG	p.S348fs	INDEL	Frameshift Insertion	2	1	YES (15.4)	NO	NO
**AML#8**	*NPM1*	*chr5:170837547*	*-/TCTG*	c.859_860insTCTG	p.W288fs*12	INDEL	Frameshift Insertion	4	11	YES (33.3)	YES (46.1)	N/E
	*CEBPA*	*chr19:33793152*	*-/G*	c.168dupC	p.E57fs	INDEL	Frameshift Insertion	1	1	YES (44.3)	NO	YES
**AML#9**	*NPM1*	*chr5:170837547*	*-/TCTG*	c.859_860insTCTG	p.W288fs*12	INDEL	Frameshift Insertion	4	11	YES (31.0)	YES (55.9)	N/E
	*CEBPA*	*chr19:33792731*	*-/GCGGGT*	c.589_590insACCCGC	p.H195_P196dup	INDEL	Nonframe shift Insertion	6	1	NO	YES (18.1)	NO
**AML#10**	*IDH2*	*chr15:90631934*	*C/T*	c.419G	p.R140	SNV	missense	1	4	YES (16.1)	YES (12.1)	N/E
**AML#13**	*CEBPA*	*chr19:33792387*	*-/CTGCGTC TCCACGTTGCGCTGCTTGGC*	c.933_934insGCCAAGCAGCGCAACGTGGAGACGCAG	p.A303_Q311dup	INDEL	Nonframe shift Insertion	27	1	YES (26.2)	YES (38.9)	N/E
	*CEBPA*	*chr19:33793082*	*-/C*	c.238dupG	p.D80fs	INDEL	Frame shift Insertion	1	1	YES (30.4)	NO	YES
**AML#14**	*CEBPA*	*chr19:33792381*	*-/CTT*	c.939_940insAAG	p.K313dup	INDEL	Nonframe shift Insertion	3	1	YES (27.1)	YES (48.7)	N/E
	*CEBPA*	*chr19:33793174*	*CG/C*	c.146delC	p.P49fs	INDEL	Frameshift Deletion	1	1	YES (42.1)	YES (57.5)	N/E
	*CEBPA*	*chr19:33792729*	*G/A*	c.592C > T	p.P198S	SNV	missense	1	1	NO	YES (16.2)	NO
**AML#15**	*CEBPA*	*chr19:33792729*	*G/A*	c.592C > T	p.P198S	SNV	missense	1	1	NO	YES (14.8)	NO
**AML#16**	*NPM1*	*chr5:170837545*	*-/TGCA*	c.861_862insTGCA	p.W288fs*12	INDEL	Frameshift Insertion	4	11	YES (47.6)	YES (46.6)	N/E
	*IDH2*	*chr15:90631934*	*C/T*	c.419G	p.R140	SNV	missense	1	4	YES (60.7)	YES (53.8)	N/E
**AML#17**	*NPM1*	*chr5:170837545*	*-/TGCA*	c.861_862insTGCA	p.W288fs*12	INDEL	Frameshift Insertion	4	11	YES (39.1)	YES (57.6)	N/E
	*FLT3*	*chr13:28592642*	*C/A*	c.2503G	p.D835	SNV	missense	1	20	YES (49.0)	YES (50.0)	N/E
**AML#18**	*TP53*	*chr17:7577120*	*C/T*	c.818G > A	p.R273H	SNV	missense	1	8	YES (78.5)	YES (72.8)	N/E
	*CEBPA*	*chr19:33792729*	*G/A*	c.592C > T	p.P198S	SNV	missense	1	1	NO	YES (15.7)	NO
**AML#19**	*TP53*	*chr17:7577538*	*C/T*	c.743G > A	p.R248Q	SNV	missense	1	7	YES (68.2)	YES (63.1)	N/E
**AML#20**	*TP53*	*chr17:7577082*	*C/T*	c.856G > A	p.E286K	SNV	missense	1	8	YES (73.1)	YES (79.1)	N/E
**AML#21**	*TP53*	*chr17:7577094*	*G/A*	c.844C > T	p.R282W	SNV	missense	1	8	YES (48.7)	YES (49.1)	N/E
	*TP53*	*chr17:7578475*	*G/C*	c.455C > G	p.P152R	SNV	missense	1	5	YES (21.0)	YES (11.5)	N/E
	*CEBPA*	*chr19:33792731*	*-/GCGGGT*	c.589_590insACCCGC	p.H195_P196dup	INDEL	Nonframe shift Insertion	6	1	NO	YES (25.2)	NO
**AML#22**	*TP53*	*chr17:7578413*	*C/T*	c.517G > A	p.V173M	SNV	missense	1	5	YES (18.1)	YES (45.3)	N/E
	*TP53*	*chr17:7579715*	*AG/A*	c.81del	p.E28Kfs * 16	INDEL	Frameshift Deletion	1	3	YES (45.0)	YES (41.8)	N/E

Note that * in Amino Acid Change column is used to indicate a stop codon, according to the nomenclature recommendations of the Human Genome Variation Society (HGVS). SNV: single nucleotide variant, INDEL: insertion/deletion, VAF: Variant Allele Frequency, N/E: not evaluated.

## Supplementary Materials

The following are available online at https://www.mdpi.com/2073-4425/10/12/1026/s1, Table S1: Clinicopathologic data for each AML case. Table S2: Depth of coverage of all amplicons for each AML case. Table S3: Depth of coverage and error rate for the variants detected (hotspot mutations and rare variants) in the negative control. Table S4: Analysis of the *CEBPA* and *TP53* variants detected in the negative control. Figure S1: Comparison between *FLT3* ITD sequences obtained from MinION sequencing (black), S5 sequencing (red) and Sanger sequencing (blue). Dots represent unmatched positions. Underlined: alternative alignments due to different ITD consensus calculations. File S1: Complete pipeline with command lines and parameters. File S2: Filtering R script.
