# Supplementary material for: Nanopore Targeted Sequencing for Rapid Gene Mutations Detection in Acute Myeloid Leukemia
Source: Genes (Basel). 2019 Dec 9;10(12):1026. doi: 10.3390/genes10121026 (PMC6947272; doi:10.3390/genes10121026)
Supplement: Supplementary file 1 [file genes-10-01026-s001.zip › Supplementary files/Supplementary File S1.docx]

#PIPELINES AND COMAND LINES FOR MINION LAM PANEL ANALYSIS

###########################################################################################################################################################################################################################################

#Variant SNV/Indels analysis

bwa mem -M path/to/reference.fasta path/to/controlHD.fastq | samtools sort -o path/to/HD.sorted.bam # map Healty donor reads

bwa mem -M path/to/reference.fasta path/to/patient.fastq | samtools sort -o path/to/patient.sorted.bam # map patient reads

samtools mpileup -f path/to/reference.fasta -q 1 -B path/to/HD.sorted.bam path/to/patient.sorted.bam > path/to/patient.mpileup

java -jar /path/to/varscan/VarScan.v2.4.3.jar somatic path/to/patient.mpileup path/to/patient_output/patientID -â€“min-coverage 10 --min-var-freq 0.05 --mpileup 1 --min-avg-qual 5 --strand-filter 0

cd path/to/patient_output

Rscript path/to/Filtering.R #Supplementary File 2

sed -e 's/,/./g' path/to/patient_output/final.snp > path/to/patient_output/final.SNV

sed -e 's/,/./g' path/to/patient_output/final.indel > path/to/patient_output/final.indel

python /Varscan2VCF/vscan2vcf.py path/to/patient_output/final.SNV > path/to/patient_output/final.SNV.vcf # Python script to convert varscan output to VCF

python /Varscan2VCF/vscan2vcf.py path/to/patient_output/final.indel > path/to/patient_output/final.indel.vcf # available at: https://github.com/student-t/Varscan2VCF

perl /annovar/table_annovar.pl path/to/patient_output/final.SNV.vcf /annovar/humandb/ -buildver hg19 -out path/to/patient_output/SNV.ANNO -remove -protocol refGene,exac03,avsnp150,cosmic81_coding,cosmic81_noncoding -operation g,f,f,f,f -nastring . -vcfinput

perl /annovar/table_annovar.pl path/to/patient_output/final.indel.vcf /annovar/humandb/ -buildver hg19 -out path/to/patient_output/final/Indel.ANNO -remove -protocol refGene,exac03,avsnp150,cosmic81_coding,cosmic81_noncoding -operation g,f,f,f,f -nastring . -vcfinput

###########################################################################################################################################################################################################################################

#FLT3/ITD Detection

/Sniffles/bin/sniffles-core-1.0.8/sniffles -m path/to/patient.sorted.bam -v path/to/patient_output/patientID.SV.vcf -f 0.1 -l 10 -n 20

/Sniffles/bin/sniffles-core-1.0.8/sniffles -m path/to/patient.sorted.bam -b path/to/patient_output/patientID.SV.bedpe -l 10

#Assembly FLT3 ITD

samtools view -b path/to/patient.sorted.bam "13:28607916-28608407" > path/to/patient.ITD.bam

samtools bam2fq path/to/patient.ITD.bam | seqtk seq -A - > path/to/patient.ITD.fasta

/CAP3/cap3 path/to/patient.ITD.fasta -p 80

###########################################################################################################################################################################################################################################
