# Supplementary material for: Nanopore Targeted Sequencing for Rapid Gene Mutations Detection in Acute Myeloid Leukemia
Source: Genes (Basel). 2019 Dec 9;10(12):1026. doi: 10.3390/genes10121026 (PMC6947272; doi:10.3390/genes10121026)
Supplement: Supplementary file 1 [file genes-10-01026-s001.zip › Supplementary files/Supplementary File S2.docx]

#Filtering.R script launched in the pipeline for MinION LAM Panel Analysis (Supplementary File S1)

#rm(list = ls())

Filenames <- list.files(getwd(), pattern = ".snp")

x<-read.table(Filenames, sep="\t", as.is=T, header=T)

normal_vaf<-NULL

for(i in 1:nrow(x)){

#print(i)

y1<-x[,7][i]<-gsub(",", ".",x[,7][i])

z1<-as.numeric(sub("%","",y1,fixed=TRUE))/100

z1<-round(z1, digits=3)

normal_vaf<-c(normal_vaf,z1)

}

tumor_vaf<-NULL

for(i in 1:nrow(x)){

#print(i)

y2<-x[,11][i]<-gsub(",", ".",x[,11][i])

z2<-as.numeric(sub("%","",y2,fixed=TRUE))/100

z2<-round(z2, digits=3)

tumor_vaf<-c(tumor_vaf,z2)

}

delta<-NULL

for(i in 1:nrow(x)){

#print(i)

diff<-(tumor_vaf[i]-normal_vaf[i])

delta<-c(delta,diff)

}

x2<-cbind(x[,1:23],normal_vaf,tumor_vaf,delta)

#write.table(x2,file="x2.txt", sep="\t",row.names=F,quote=F)

data2<-x2[(x2$delta>0.1)==T,]

#dim(data2)

data3<-data2[(data2$normal_vaf<0.07)==T,]

data4<-data3[(data3$somatic_p_value<0.01)==T,]

data4<-data4[,-c(24:26)]

write.table(data4,file="final_snp.txt",sep="\t",row.names=F,quote=F)

Filenames <- list.files(getwd(), pattern = ".indel")

x<-read.table(Filenames, sep="\t", as.is=T, header=T)

normal_vaf<-NULL

for(i in 1:nrow(x)){

#print(i)

y1<-x[,7][i]<-gsub(",", ".",x[,7][i])

z1<-as.numeric(sub("%","",y1,fixed=TRUE))/100

z1<-round(z1, digits=3)

normal_vaf<-c(normal_vaf,z1)

}

tumor_vaf<-NULL

for(i in 1:nrow(x)){

#print(i)

y2<-x[,11][i]<-gsub(",", ".",x[,11][i])

z2<-as.numeric(sub("%","",y2,fixed=TRUE))/100

z2<-round(z2, digits=3)

tumor_vaf<-c(tumor_vaf,z2)

}

delta<-NULL

for(i in 1:nrow(x)){

#print(i)

diff<-(tumor_vaf[i]-normal_vaf[i])

delta<-c(delta,diff)

}

x2<-cbind(x[,1:23],normal_vaf,tumor_vaf,delta)

#write.table(x2,file="x2.txt", sep="\t",row.names=F,quote=F)

data2<-x2[(x2$delta>0.1)==T,]

dim(data2)

data3<-data2[(data2$normal_vaf<0.07)==T,]

data4<-data3[(data3$somatic_p_value<0.01)==T,]

data4<-data4[,-c(24:26)]

write.table(data4,file="final_indel.txt",sep="\t",row.names=F,quote=F)
