# Supplementary material for: Nanopore Targeted Sequencing for Rapid Gene Mutations Detection in Acute Myeloid Leukemia
Source: Genes (Basel). 2019 Dec 9;10(12):1026. doi: 10.3390/genes10121026 (PMC6947272; doi:10.3390/genes10121026)
Supplement: Supplementary file 1 [file genes-10-01026-s001.zip › Supplementary files/Supplementary Table S1.docx]

| **Case** | **WHO diagnosis** | **Bone marrow blast count (%)** | **Karyotype** | **Bone marrow aspirate cellularity** |
| --- | --- | --- | --- | --- |
| **AML#1** | AML NOS | >50 | 46,XY[20] | +++ |
| **AML#2** | AML NOS | >50 | 46,XY[8] | +++ |
| **AML#3** | AML with mutated *NPM1* | >50 | 46,XY[20] | +++ |
| **AML#4** | AML NOS | >80 | 46,XY,add(7p)[3] / 46,XY[2] | +++ |
| **AML#5** | AML with mutated *NPM1* | >50 | N/A | +++ |
| **AML#6** | AML NOS | >50 | 46,XX[20] | +++ |
| **AML#7** | AML with mutated *NPM1* | >50 | 46,XY[20] | ++ |
| **AML#8** | AML with mutated *NPM1* | >50 | 46,XX[12] | +++ |
| **AML#9** | AML with mutated *NPM1* | >50 | N/A | +++ |
| **AML#10** | AML NOS | >50 | 46,XY,add(7q)[20] | ++ |
| **AML#11** | AML NOS | >80 | 46,XX,del(5q),-17,i(17q),+m[2] / 41~45,XX,-X, trc(1;12;16)(p11;q24.3p13;q24),del(1p),del(2p), -2,add(3q),del(5q),-7,del(7q),add(11p),dic(12;16), (p13;q24),-12,-16,add(16q),-17,-17,-21,+1~5mar[cp8] | +++ |
| **AML#12** | AML NOS | >50 | N/A | +++ |
| **AML#13** | AML with biallelic mutations of *CEBPA* | >50 | N/A | +++ |
| **AML#14** | AML with biallelic mutations of *CEBPA* | >50 | N/A | +++ |
| **AML#15** | AML NOS | >50 | N/A | +++ |
| **AML#16** | AML with mutated *NPM1* | >50 | 46,XX[20] | ++ |
| **AML#17** | AML with mutated *NPM1* | >50 | 46,XX[20] | +++ |
| **AML#18** | AML NOS | >50 | 43,XY,del(5q),?del(15q),-17,-18,-18,?add(22q)[4] / 44,XY,idem,+mar[5] / 46,XY[7] | +++ |
| **AML#19** | AML NOS | >80 | 46,XY,del(7q),del(20q)[2] / 46~50,XY,add(4p),del(7q),  -9,add(17p),del(20q),+1~5 mar[cp8] / 46,XY[2] | +++ |
| **AML#20** | AML NOS | >50 | 45,XX,-3,add(5q),-7,add(8p),-12,inv(12)(q15q24.1), ?del(14q),-17,+mar1,+mar2,+mar3[15] | ++ |
| **AML#21** | AML NOS | >50 | 43,XY,del(5q),  -6,del(7q),der(11)t(6;11)(p?25;p?15)ins(11;?)(p?15;?), dic(12;17)(p?12;p?13),-20[13] / 46,XY[2] | +++ |
| **AML#22** | AML NOS | >80 | 45,XY,-2,der(5)add(5p)del(5q),del(7q),add(17p),  -17,+mar1[15] /  45,XY,-2,der(5)add(5p)del(5q),del(7q),-17,  -17,+mar1,+mar2[3] / 46,XY[2] | +++ |

Clinicopathologic data for each AML case. NOS: not otherwise specified, N/A: not available, +: sufficient, ++: good, +++: excellent.
