# Supplementary material for: Nanopore Targeted Sequencing for Rapid Gene Mutations Detection in Acute Myeloid Leukemia
Source: Genes (Basel). 2019 Dec 9;10(12):1026. doi: 10.3390/genes10121026 (PMC6947272; doi:10.3390/genes10121026)
Supplement: Supplementary file 1 [file genes-10-01026-s001.zip › Supplementary files/Supplementary Table S3.docx]

|  | ***IDH1 chr2:209113113 G/A* (p.R132)** | |
| --- | --- | --- |
|  | **read depth** | **error rate (%)** |
| **NC1** | 6322 | 0.70 |
| **NC2** | 3656 | 0.74 |
|  |  |  |
| **MEAN** | 4989 | 0.72 |
|  |  |  |
|  | ***IDH2 chr15:90631934 C/T* (p.R140)** | |
|  | **read depth** | **error rate (%)** |
| **NC1** | 4236 | 2.43 |
| **NC2** | 1941 | 1.39 |
|  |  |  |
| **MEAN** | 3088.5 | 1.91 |
|  |  |  |
|  | ***IDH2 chr15:90631934 C/A* (p.R140)** | |
|  | **read depth** | **error rate (%)** |
| **NC1** | 4236 | 0.99 |
| **NC2** | 1941 | 1.08 |
|  |  |  |
| **MEAN** | 3088.5 | 1.04 |
|  |  |  |
|  | ***NPM1 chr5:170837547 -/TCTG* (p.W288fs*12)** | |
|  | **read depth** | **error rate (%)** |
| **NC1** | 197 | 0.51 |
| **NC2** | 150 | 1.33 |
|  |  |  |
| **MEAN** | 173.5 | 0.92 |
|  |  |  |
|  | ***NPM1 chr5:170837545 -/TGCA* (p.W288fs*12)** | |
|  | **read depth** | **error rate (%)** |
| **NC1** | 197 | 6.60 |
| **NC2** | 150 | 7.33 |
|  |  |  |
| **MEAN** | 173.5 | 6.97 |
|  |  |  |
|  | ***FLT3 chr13:28592642 C/A* (p.D835)** | |
|  | **read depth** | **error rate (%)** |
| **NC1** | 8950 | 0.34 |
| **NC2** | 3272 | 0.55 |
|  |  |  |
| **MEAN** | 6111 | 0.44 |
|  |  |  |
|  | ***FLT3 chr13:28608329 A/C* (p.L576R)** | |
|  | **read depth** | **error rate (%)** |
| **NC1** | 1813 | 0.28 |
| **NC2** | 1322 | 0.15 |
|  |  |  |
| **MEAN** | 1567.5 | 0.21 |
|  |  |  |
|  | ***CEBPA chr19:33793152 -/G* (p.E57fs)** | |
|  | **read depth** | **error rate (%)** |
| **NC1** | 1468 | 6.06 |
| **NC2** | 1095 | 4.84 |
|  |  |  |
| **MEAN** | 1281.5 | 5.45 |
|  |  |  |
|  | ***CEBPA chr19:33792387 -/CTGCGTCTCCACGTTGCGCTGCTTGGC* (p.A303_Q311dup)** | |
|  | **read depth** | **error rate (%)** |
| **NC1** | 1480 | 0.00 |
| **NC2** | 1122 | 0.00 |
|  |  |  |
| **MEAN** | 1301 | 0.00 |
|  |  |  |
|  | ***CEBPA chr19:33793082 -/C* (p.D80fs)** | |
|  | **read depth** | **error rate (%)** |
| **NC1** | 1477 | 2.10 |
| **NC2** | 1102 | 1.00 |
|  |  |  |
| **MEAN** | 1289.5 | 1.55 |
|  |  |  |
|  | ***CEBPA chr19:33792381 -/CTT* (p.K313dup)** | |
|  | **read depth** | **error rate (%)** |
| **NC1** | 1476 | 2.64 |
| **NC2** | 1122 | 3.03 |
|  |  |  |
| **MEAN** | 1299 | 2.84 |
|  |  |  |
|  | ***CEBPA chr19:33793174 CG/C* (p.P49fs)** | |
|  | **read depth** | **error rate (%)** |
| **NC1** | 1462 | 12.18 |
| **NC2** | 1092 | 12.91 |
|  |  |  |
| **MEAN** | 1277 | 12.54 |
|  |  |  |
|  | ***TP53 chr17:7577120 C/T* (p.R273H)** | |
|  | **read depth** | **error rate (%)** |
| **NC1** | 11863 | 2.88 |
| **NC2** | 7716 | 2.70 |
|  |  |  |
| **MEAN** | 9789.5 | 2.79 |
|  |  |  |
|  | ***TP53 chr17:7577538 C/T* (p.R248Q)** | |
|  | **read depth** | **error rate (%)** |
| **NC1** | 10618 | 1.22 |
| **NC2** | 7163 | 1.23 |
|  |  |  |
| **MEAN** | 8890.5 | 1.23 |
|  |  |  |
|  | ***TP53 chr17:7577082 C/T* (p.E286K)** | |
|  | **read depth** | **error rate (%)** |
| **NC1** | 11824 | 2.05 |
| **NC2** | 7714 | 2.42 |
|  |  |  |
| **MEAN** | 9769 | 2.24 |
|  |  |  |
|  | ***TP53 chr17:7577094 G/A* (p.R282W)** | |
|  | **read depth** | **error rate (%)** |
| **NC1** | 11830 | 11.55 |
| **NC2** | 7716 | 12.07 |
|  |  |  |
| **MEAN** | 9773 | 11.81 |
|  |  |  |
|  | ***TP53 chr17:7578475 G/C* (p.P152R)** | |
|  | **read depth** | **error rate (%)** |
| **NC1** | 4258 | 4.20 |
| **NC2** | 1850 | 4.49 |
|  |  |  |
| **MEAN** | 3054 | 4.35 |
|  |  |  |
|  | ***TP53 chr17:7578413 C/T* (p.V173M)** | |
|  | **read depth** | **error rate (%)** |
| **NC1** | 4313 | 1.74 |
| **NC2** | 1863 | 2.04 |
|  |  |  |
| **MEAN** | 3088 | 1.89 |
|  |  |  |
|  | ***TP53 chr17:7579715 AG/A* (p.E28Kfs*16)** | |
|  | **read depth** | **error rate (%)** |
| **NC1** | 6424 | 10.96 |
| **NC2** | 4813 | 9.54 |
|  |  |  |
| **MEAN** | 5618.5 | 10.25 |

Depth of coverage and error rate for the variants detected (hotspot mutations and rare variants) in the negative control. NC1: negative control sequenced in run 1, NC2: negative control sequenced in run 2.
