# Supplementary material for: Nanopore Targeted Sequencing for Rapid Gene Mutations Detection in Acute Myeloid Leukemia
Source: Genes (Basel). 2019 Dec 9;10(12):1026. doi: 10.3390/genes10121026 (PMC6947272; doi:10.3390/genes10121026)
Supplement: Supplementary file 1 [file genes-10-01026-s001.zip › Supplementary files/Supplementary Table S4.docx]

| **Chromosome** | **Start** | **End** | **Genotype** | **NC1** | **NC2** | **VAF (%)** | **Location** | **Gene** |
| --- | --- | --- | --- | --- | --- | --- | --- | --- |
| *17* | *7572997* | *7572997* | *G/A* | no | yes | 0.1064 | exonic | *TP53* |
| *19* | *33793332* | *33793332* | *A/G* | yes | no | 0.1073 | exonic | *CEBPA* |
| *17* | *7572991* | *7572991* | *T/C* | yes | no | 0.112 | exonic | *TP53* |
| *19* | *33792809* | *33792809* | *T/C* | yes | yes | 0.1213 | exonic | *CEBPA* |
| *19* | *33792806* | *33792806* | *T/C* | yes | no | 0.1231 | exonic | *CEBPA* |
| *19* | *33792560* | *33792560* | *T/C* | yes | yes | 0.1282 | exonic | *CEBPA* |
| *19* | *33792338* | *33792338* | *A/C* | yes | no | 0.137 | exonic | *CEBPA* |
| *19* | *33792810* | *33792810* | *T/C* | no | yes | 0.1383 | exonic | *CEBPA* |
| *19* | *33792753* | *33792753* | *A/G* | yes | yes | 0.1677 | exonic | *CEBPA* |
| *17* | *7576524* | *7576524* | *C/T* | yes | yes | 0.1728 | splicing | *TP53* |
| *19* | *33792813* | *33792813* | *C/T* | yes | yes | 0.1774 | exonic | *CEBPA* |
| *17* | *7572962* | *7572962* | *GT/G* | yes | yes | 0.6375 | exonic | *TP53* |
| *17* | *7572990* | *7572990* | *CT/C* | yes | yes | 0.3098 | exonic | *TP53* |
| *17* | *7573943* | *7573943* | *TC/T* | yes | yes | 0.5027 | exonic | *TP53* |
| *17* | *7574012* | *7574012* | *-/A* | yes | yes | 0.2226 | exonic | *TP53* |
| *17* | *7576636* | *7576636* | *CT/C* | yes | yes | 0.155 | exonic | *TP53* |
| *17* | *7576896* | *7576896* | *TG/T* | yes | yes | 0.2429 | exonic | *TP53* |
| *17* | *7577031* | *7577031* | *TC/T* | yes | yes | 0.3311 | exonic | *TP53* |
| *17* | *7577035* | *7577035* | *TG/T* | yes | yes | 0.1682 | exonic | *TP53* |
| *17* | *7577057* | *7577057* | *TC/T* | yes | yes | 0.2104 | exonic | *TP53* |
| *17* | *7577127* | *7577127* | *CA/C* | yes | no | 0.1527 | exonic | *TP53* |
| *17* | *7577508* | *7577508* | *TC/T* | yes | yes | 0.1779 | exonic | *TP53* |
| *17* | *7577536* | *7577536* | *-/C* | yes | yes | 0.2373 | exonic | *TP53* |
| *17* | *7578191* | *7578191* | *AG/A* | yes | yes | 0.1833 | exonic | *TP53* |
| *17* | *7578279* | *7578279* | *AGG/A* | yes | yes | 0.3127 | exonic | *TP53* |
| *17* | *7578397* | *7578397* | *TG/G* | yes | yes | 0.2654 | exonic | *TP53* |
| *17* | *7578474* | *7578474* | *CG/C* | yes | yes | 0.3393 | exonic | *TP53* |
| *17* | *7578546* | *7578546* | *AG/A* | yes | no | 0.1615 | exonic | *TP53* |
| *17* | *7579382* | *7579382* | *GT/G* | yes | yes | 0.2007 | exonic | *TP53* |
| *17* | *7579419* | *7579419* | *AG/A* | yes | yes | 0.3557 | exonic | *TP53* |
| *17* | *7579432* | *7579432* | *AG/A* | yes | no | 0.1607 | exonic | *TP53* |
| *17* | *7579470* | *7579470* | *CG/C* | yes | yes | 0.2336 | exonic | *TP53* |
| *17* | *7579507* | *7579507* | *TG/T* | yes | yes | 0.1717 | exonic | *TP53* |
| *17* | *7579584* | *7579584* | *AG/A* | yes | yes | 0.4334 | exonic | *TP53* |
| *17* | *7579874* | *7579874* | *AGGG/A* | yes | yes | 0.4212 | exonic | *TP53* |
| *17* | *7579889* | *7579889* | *AG/A* | yes | yes | 0.5199 | exonic | *TP53* |
| *19* | *33792271* | *33792271* | *CA/C* | yes | no | 0.1516 | exonic | *CEBPA* |
| *19* | *33792781* | *33792781* | *AG/A* | yes | yes | 0.2232 | exonic | *CEBPA* |
| *19* | *33792808* | *33792808* | *CT/C* | yes | yes | 0.3444 | exonic | *CEBPA* |
| *19* | *33792884* | *33792884* | *AG/A* | yes | yes | 0.2679 | exonic | *CEBPA* |
| *19* | *33792937* | *33792937* | *CG/C* | yes | no | 0.1559 | exonic | *CEBPA* |
| *19* | *33792952* | *33792952* | *TC/T* | yes | yes | 0.6802 | exonic | *CEBPA* |
| *19* | *33792979* | *33792979* | *GC/G* | yes | yes | 0.204 | exonic | *CEBPA* |
| *19* | *33792991* | *33792991* | *GC/G* | yes | no | 0.1733 | exonic | *CEBPA* |
| *19* | *33793252* | *33793252* | *CG/G* | yes | yes | 0.5489 | exonic | *CEBPA* |
| *19* | *33793279* | *33793279* | *CG/C* | yes | yes | 0.301 | exonic | *CEBPA* |
| *19* | *33793320* | *33793320* | *TG/T* | yes | yes | 0.483 | exonic | *CEBPA* |
| *19* | *33793330* | *33793330* | *AG/A* | yes | yes | 0.1587 | exonic | *CEBPA* |
| *17* | *7574002* | *7574002* | *-/A* | no | yes | 0.1804 | exonic | *TP53* |
| *19* | *33792374* | *33792374* | *TC/T* | no | yes | 0.1619 | exonic | *CEBPA* |
| *19* | *33792537* | *33792537* | *CG/C* | no | yes | 0.1724 | exonic | *CEBPA* |
| *19* | *33792902* | *33792902* | *TC/T* | no | yes | 0.189 | exonic | *CEBPA* |

Analysis of the *CEBPA* and *TP53* variants detected in the negative control. NC1: negative control sequenced in run 1, NC2: negative control sequenced in run 2, VAF: variant allele frequency.
