# Supplementary figures and images for: Nanopore Targeted Sequencing for Rapid Gene Mutations Detection in Acute Myeloid Leukemia
Source: Genes (Basel). 2019 Dec 9;10(12):1026. doi: 10.3390/genes10121026 (PMC6947272; doi:10.3390/genes10121026)

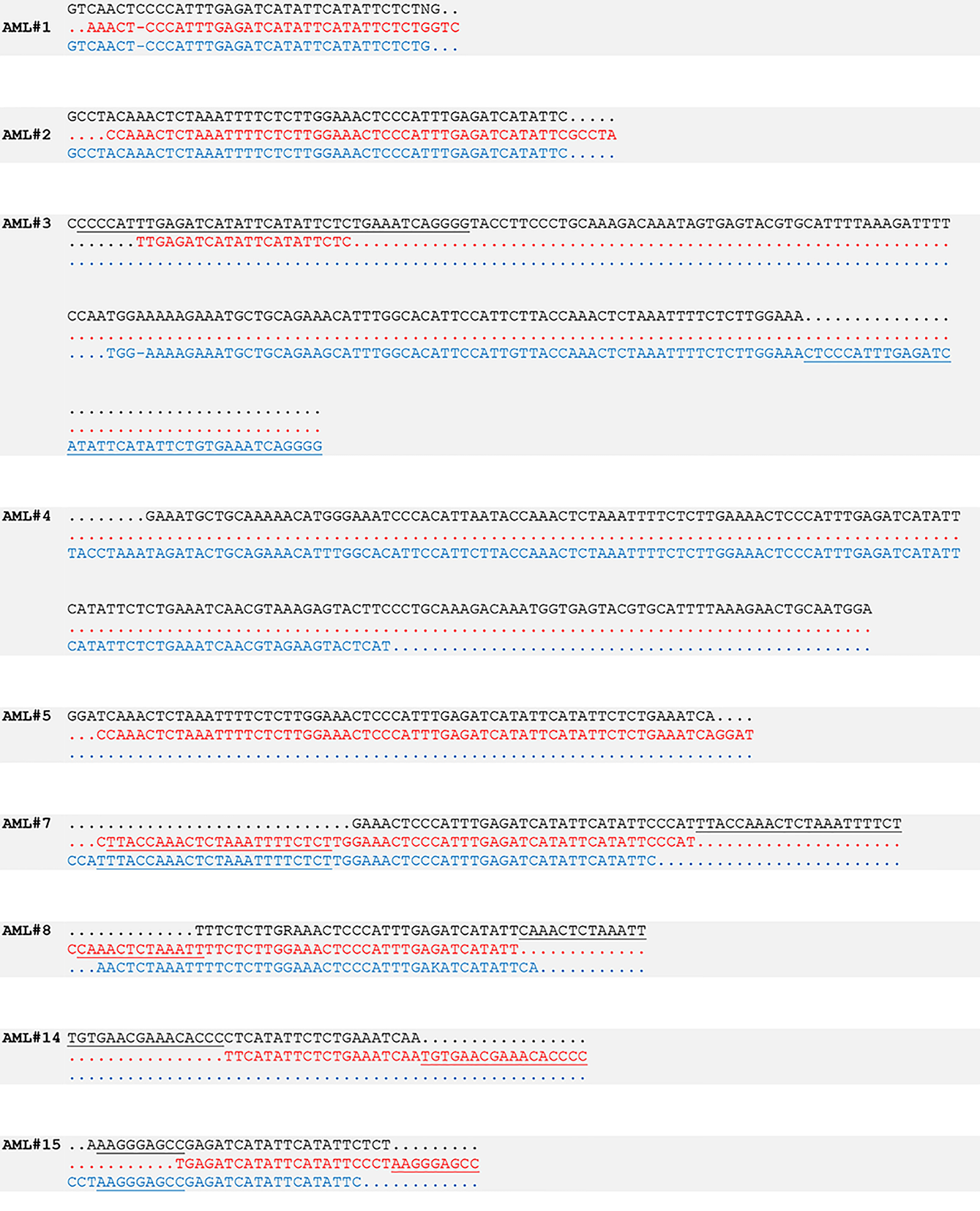

Supplement: Supplementary file 1 [file genes-10-01026-s001.zip › Supplementary files/Supplementary Figure S1.tif]
