# Supplementary material for: Nanopore Targeted Sequencing for Rapid Gene Mutations Detection in Acute Myeloid Leukemia
Source: Genes (Basel). 2019 Dec 9;10(12):1026. doi: 10.3390/genes10121026 (PMC6947272; doi:10.3390/genes10121026)
Supplement: Supplementary file 1 [file genes-10-01026-s001.zip › Supplementary files/Supplementary Table S2.doc]

|  | ***CEBPA ex1*** | ***FLT3 ex14-15*** | ***FLT3 ex20*** | ***IDH1 ex4*** | ***IDH2 ex4*** | ***NPM1 ex11*** | ***TP53 ex2-4*** | ***TP53 ex5-6*** | ***TP53 ex7-9*** | ***TP53 ex10-11*** |
| --- | --- | --- | --- | --- | --- | --- | --- | --- | --- | --- |
| **AML#1** | 2310,41 | 2597,48 | 10770,04 | 4948,79 | 3294,51 | 84,08 | 8615,1 | 4679,69 | 13620,15 | 7257,48 |
| **AML#2** | 3779,18 | 5279,46 | 15000,05 | 5765,57 | 3704,66 | 190,08 | 15346,96 | 6879,86 | 16640,9 | 8647,78 |
| **AML#3** | 1011,14 | 1197,69 | 5452,72 | 3063,32 | 2183,03 | 77,12 | 4811,16 | 2332,9 | 8170,65 | 4259,99 |
| **AML#4** | 1778,38 | 1289,29 | 5765,83 | 2788,44 | 1577,44 | 41,39 | 8000,33 | 2710,03 | 10301,97 | 4969,98 |
| **AML#5** | 2649,18 | 3437,75 | 8744,82 | 6458,05 | 3680,27 | 213,5 | 11964,07 | 5597,5 | 14134,51 | 7206,22 |
| **AML#6** | 2927,35 | 2767,57 | 7574,73 | 4854,45 | 3225,23 | 140,58 | 9251,77 | 4665,08 | 13496,14 | 6298,73 |
| **AML#7** | 823,95 | 745,6 | 4848,16 | 2693,47 | 1527,99 | 19,82 | 3956,13 | 1860,95 | 6406,34 | 3308,87 |
| **AML#8** | 6577,69 | 5538,51 | 20360,18 | 4560,09 | 6441,46 | 242,93 | 25636,31 | 6110,29 | 21911,8 | 10359,82 |
| **AML#9** | 4744,53 | 2867,09 | 18679,79 | 8014,09 | 6306,84 | 243,13 | 16816,45 | 5717,11 | 18927,65 | 9118,93 |
| **AML#10** | 3010,69 | 3049,14 | 11081,71 | 4961,42 | 4175,84 | 98,99 | 12056,19 | 5514,77 | 15672,44 | 6941,51 |
| **AML#11** | 3138,16 | 2608,51 | 6937,79 | 15413,3 | 2561,21 | 109,25 | 5966,24 | 6546,53 | 10478,86 | 5404,3 |
| **AML#12** | 1860,11 | 3254,45 | 5698,32 | 5834,01 | 3106,29 | 157,14 | 5839,84 | 4263,71 | 9444,08 | 5380,87 |
| **AML#13** | 3272,75 | 4314,08 | 10229,81 | 5097,73 | 4701,03 | 155,29 | 12689,84 | 4952,16 | 13210,06 | 6784,7 |
| **AML#14** | 1072,63 | 895,18 | 3951,09 | 2420,83 | 1829,22 | 40,91 | 4679,05 | 1665,02 | 7549,02 | 3852,57 |
| **AML#15** | 1573,1 | 2215,43 | 3370,08 | 3026,1 | 2410,08 | 172,15 | 6755,92 | 2118,06 | 7558,83 | 3518 |
| **AML#16** | 1939,28 | 1980,18 | 5440,53 | 4347,3 | 2352,02 | 69,52 | 7672,93 | 3292,9 | 8563,95 | 4975,26 |
| **AML#17** | 1833,35 | 2748,06 | 4094,89 | 3564,2 | 2340,03 | 151,61 | 7440,29 | 3326,52 | 8189,92 | 3843,37 |
| **AML#18** | 798,97 | 861,82 | 2122,48 | 1855,36 | 1430,83 | 24,11 | 3242,82 | 864,88 | 4522,65 | 2200,51 |
| **AML#19** | 5675,8 | 5949,1 | 9744,63 | 4818,22 | 4386,9 | 231,74 | 14630,37 | 3542,56 | 14012,13 | 5951,89 |
| **AML#20** | 5073,49 | 3389,65 | 8377,15 | 6421,4 | 3864,09 | 123,8 | 10287,33 | 4134,98 | 11414,26 | 5058,62 |
| **AML#21** | 2488,36 | 1901,28 | 7719,18 | 4200,44 | 2864,8 | 91,59 | 8386,14 | 2880,69 | 11134,39 | 4654,88 |
| **AML#22** | 1977,09 | 1751,47 | 4085,02 | 2678,09 | 2771,88 | 120,36 | 7300,56 | 2460,81 | 10335,11 | 4422,6 |
| **Mean** | 2625,15 | 2662,29 | 7939,17 | 4916,76 | 3202,45 | 129,36 | 9292,08 | 3843,96 | 11495,42 | 5605,70 |

Depth of coverage of all amplicons for each AML case.
